# Supplementary material for: MicroRNA miR-23a cluster promotes osteocyte differentiation by regulating TGF-β signalling in osteoblasts
Source: Nat Commun. 2017 Apr 11;8:15000. doi: 10.1038/ncomms15000 (PMC5394267; doi:10.1038/ncomms15000)
Supplement: Supplementary Information — Supplementary Figures and Supplementary Tables [file ncomms15000-s1.pdf]

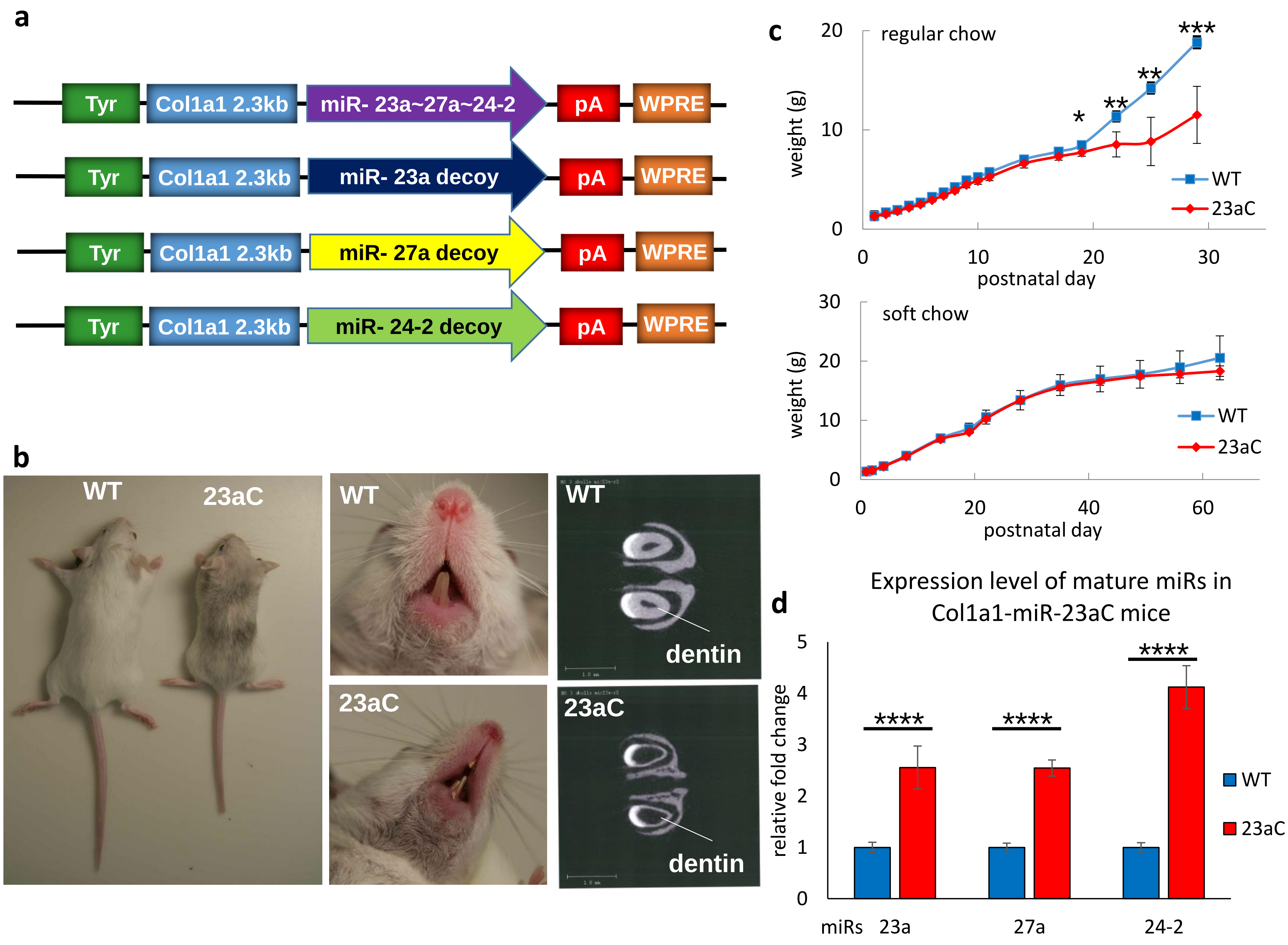
a
Tyr
Col1a1 2.3kb
miR- 23a~27a~24-2
pA
WPRE
miR- 23a decoy
miR- 27a decoy
miR- 24-2 decoy
b
WT
23aC
dentin
c
regular chow
weight (g)
postnatal day
soft chow
d
Expression level of mature miRs in Col1a1-miR-23aC mice
relative fold change
miRs
23a
27a
24-2

Supplementary Figure 1 | Osteoblast specific gain-of-function (GOF) and loss-of-function (LOF) mouse models.

a. Diagram for generating the GOF (*Col1a1-miR-23aC*) mice and the LOF (*Col1a1-miR-23a* decoy, *Col1a1-miR-27a* decoy and *Col1a1-miR-24-2* decoy) mice. The transgenes are driven by the 2.3 kb Collagen type I, alpha 1, 2.3 kb promoter (Col1a1 2.3kb) specifically in osteoblasts and are followed by polyA signal (pA). The Tyrosinase mini gene (Tyr) enables rapid genotyping by eye and fur color selection. The woodchuck posttranscriptional regulatory element (WPRE) sequence increases the expression level of the transgene and is used for PCR genotyping.

b. Representative images of 7-week-old female GOF *Col1a1-miR-23aC* (23aC) and wild-type (WT) mice. The *Col1a1-miR-23aC* mice were significantly smaller than their WT littermates due to fragility of the incisors. The dentin of the incisors was thinner in *Col1a1-miR-23aC* mice.

c. Growth curve of the *Col1a1-miR-23aC* (23aC) mice fed with either regular chow or soft chow. The fragility of the incisors hindered nutrient intake in *Col1a1-miR-23aC* mice and caused growth retardation. The weights could be normalized to that of WT littermates by supplementing with a soft chow diet. N=3 for WT, N=7 for 23aC with regular chow and N=4 for WT, N=6 for 23aC with soft chow.

d. Expression level of each microRNA in postnatal day 3 (P3) calvarial bones of WT and *Col1a1-miR-23aC* mice (N=4).

Statistical analyses used t-test and results are shown as mean ± S.D.; *P<0.05, **P<0.01, ***P<0.005, ****P<0.001.

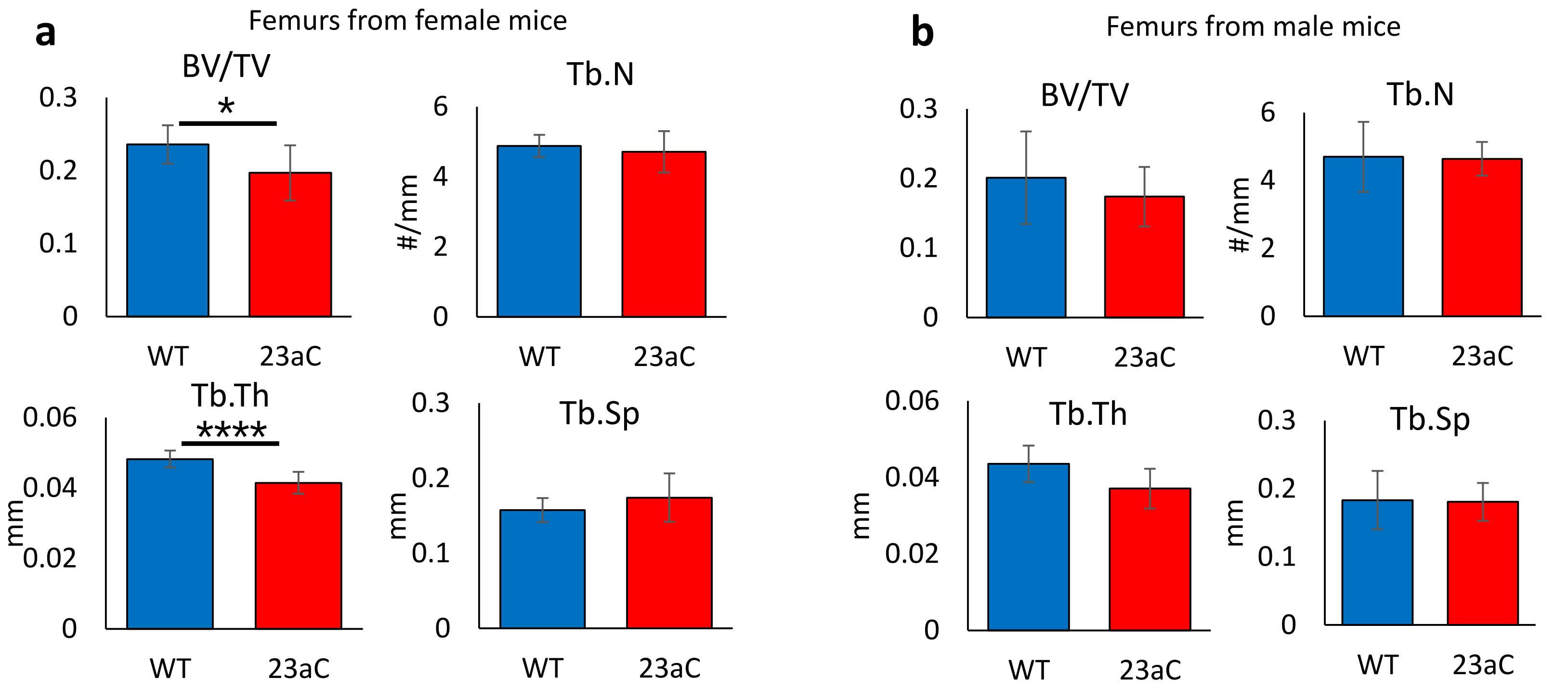
a
Femurs from female mice
BV/TV
*
0.3
0.2
0.1
0
WT
23aC
Tb.N
6
4
2
0
#/mm
WT
23aC
Tb.Th
****
0.06
0.04
0.02
0
mm
WT
23aC
Tb.Sp
0.3
0.2
0.1
0
mm
WT
23aC
b
Femurs from male mice
BV/TV
0.3
0.2
0.1
0
WT
23aC
Tb.N
6
4
2
0
#/mm
WT
23aC
Tb.Th
0.06
0.04
0.02
0
mm
WT
23aC
Tb.Sp
0.3
0.2
0.1
0
mm
WT
23aC

Supplementary Figure 2 | Additional femur µCT analysis of the GOF mouse model.

µCT analysis of trabecular bone volume/total bone volume (BV/TV), trabecular bone thickness (Tb.Th), trabecular bone number (Tb.N), trabecular bone separation (Tb.Sp) in femurs of 3-month-old **(a)** female mice (N=7 for WT, N=8 for 23aC) and **(b)** male mice (N=8 for WT, N=5 for 23aC).

Statistical analyses used t-test and results are shown as mean ± S.D.; *P<0.05, ****P<0.001.

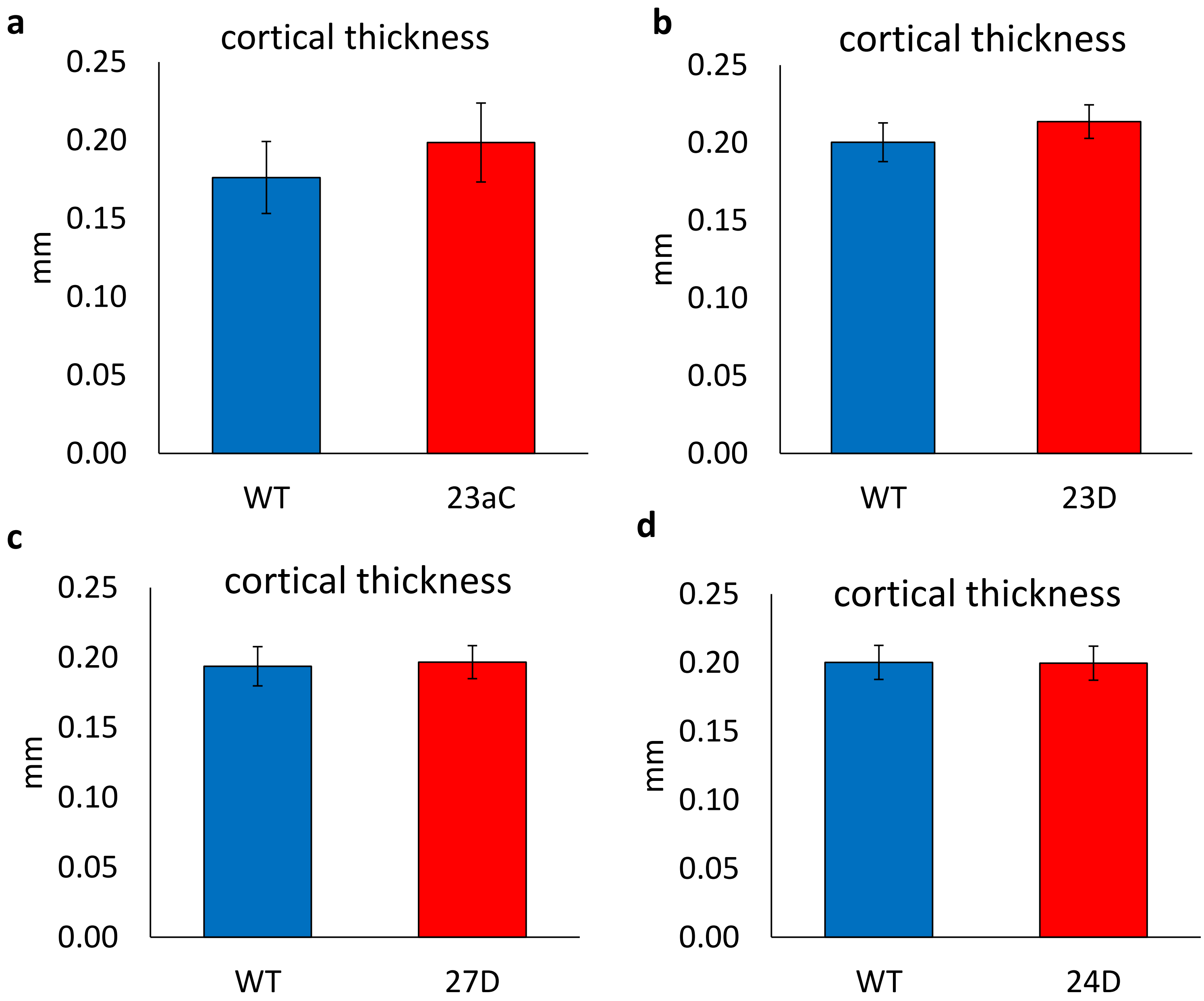
a
cortical thickness
0.25
0.20
0.15
0.10
0.05
0.00
mm
WT
23aC
b
cortical thickness
0.25
0.20
0.15
0.10
0.05
0.00
mm
WT
23D
c
cortical thickness
0.25
0.20
0.15
0.10
0.05
0.00
mm
WT
27D
d
cortical thickness
0.25
0.20
0.15
0.10
0.05
0.00
mm
WT
24D

Supplementary Figure 3 | μCT analysis of the femur cortical thickness of transgenic mouse lines.

μCT analysis of femur cortical thickness of 3-month-old female **(a)** *Col1a1-miR-23aC* (23aC), **(b)** *Col1a1-miR-23a* decoy (23D), **(c)** *Col1a1-miR-27a* decoy (27D) and **(d)** *Col1a1-miR-24-2* decoy (24D) mice (N= 7 for each group).

Statistical analyses used t-test and results are shown as mean ± S.D.; no statistically significant difference was found for any mutant line..

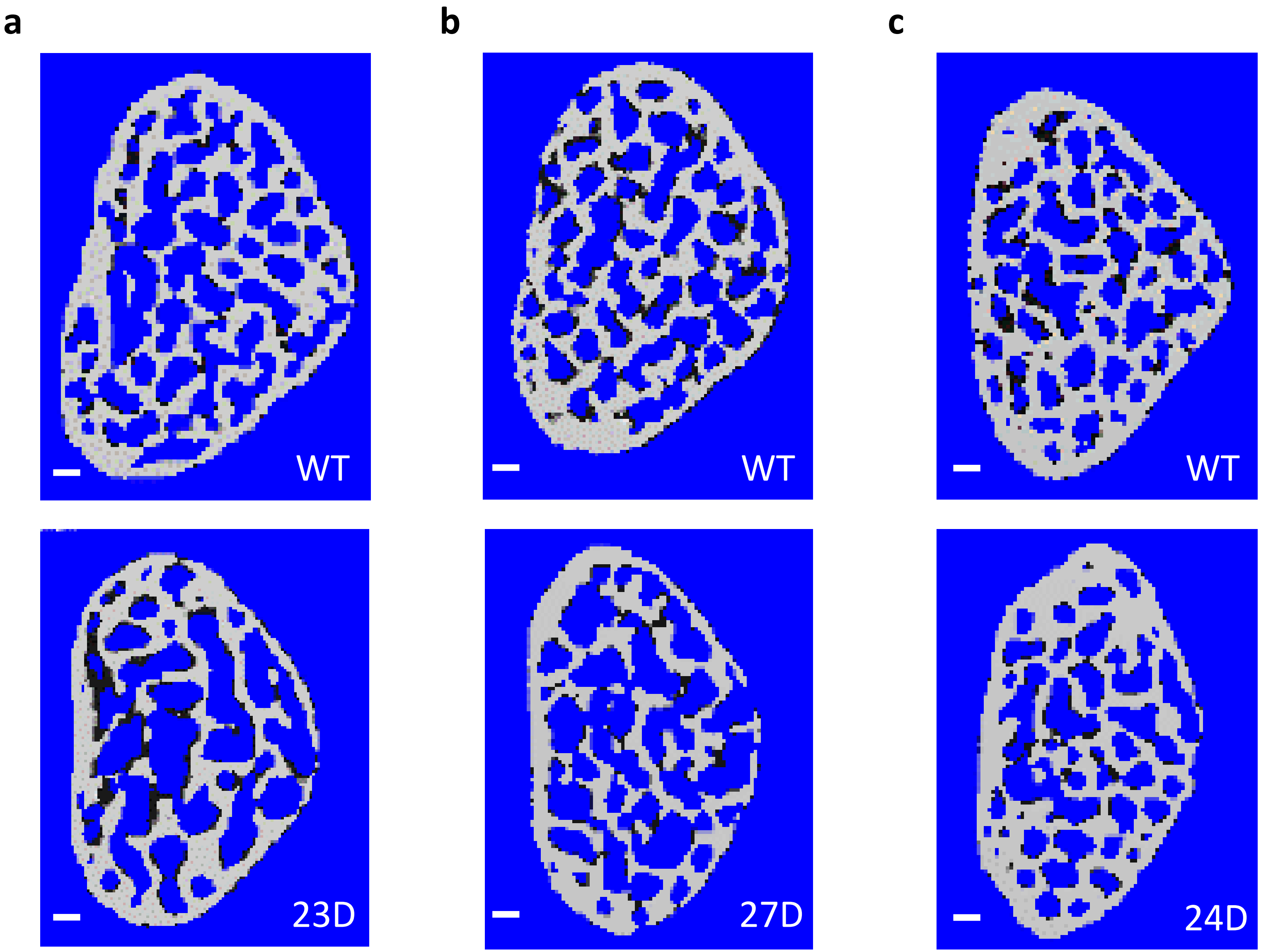
a
b
c
WT
WT
WT
23D
27D
24D

Supplementary Figure 4 | spine μCT images of LOF mouse models.

Representative μCT images of lumbar spines of **(a)** *Col1a1-miR-23a* decoy mice (23D), **(b)** *Col1a1-miR-27a* decoy mice (27D), **(c)** *Col1a1-miR-24-2* decoy mice (24D) and wild-type littermate (WT) (3-month-old females). White bars indicate scale of 100 μm.

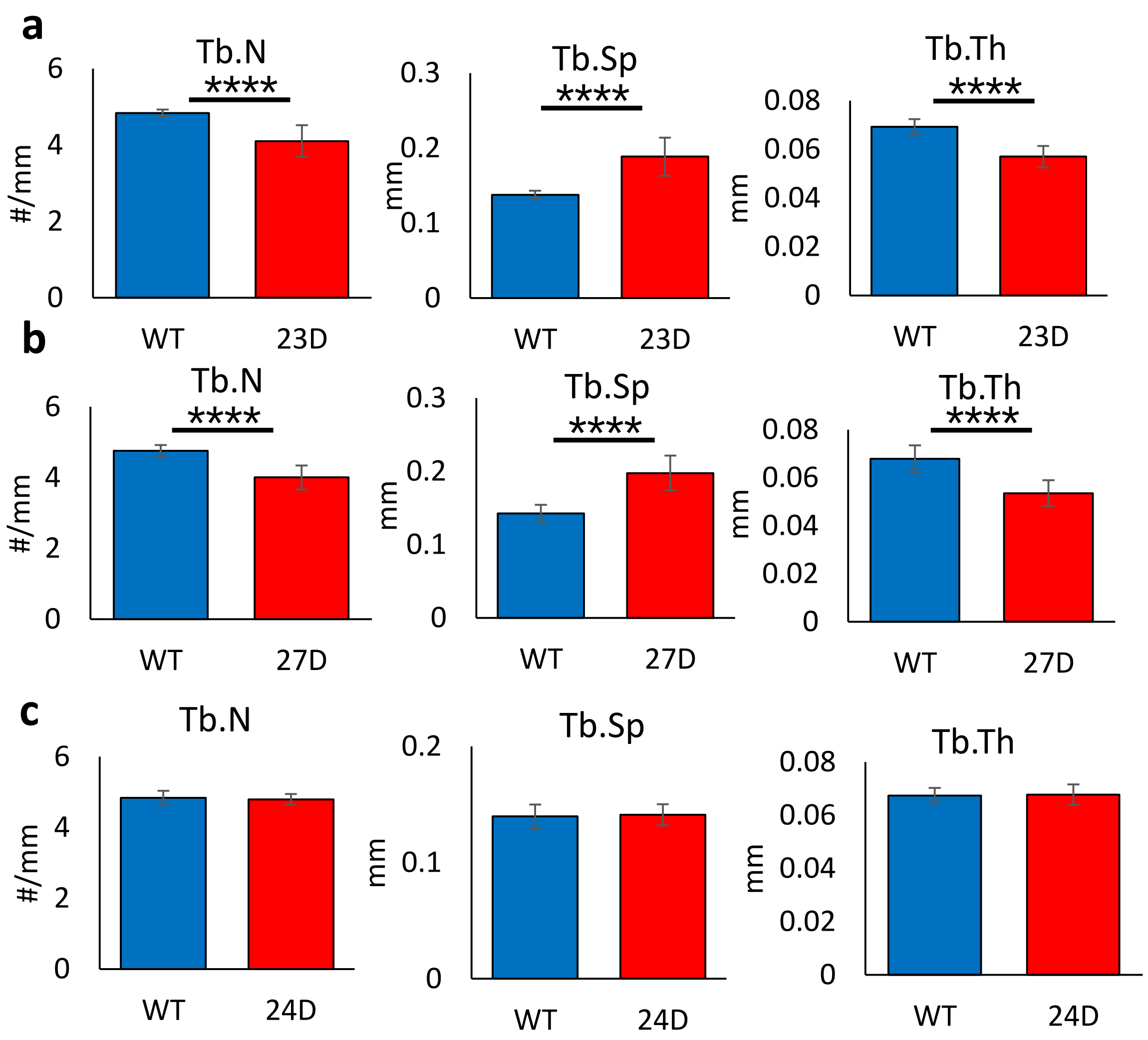
a
Tb.N
****
#/mm
6
4
2
0
WT
23D
Tb.Sp
****
mm
0.3
0.2
0.1
0
WT
23D
Tb.Th
****
mm
0.08
0.06
0.04
0.02
0
WT
23D
b
Tb.N
****
#/mm
6
4
2
0
WT
27D
Tb.Sp
****
mm
0.3
0.2
0.1
0
WT
27D
Tb.Th
****
mm
0.08
0.06
0.04
0.02
0
WT
27D
c
Tb.N
#/mm
6
4
2
0
WT
24D
Tb.Sp
mm
0.2
0.1
0
WT
24D
Tb.Th
mm
0.08
0.06
0.04
0.02
0
WT
24D

Supplementary Figure 5 | Additional spine μCT analysis of LOF mouse models.

μCT analysis of Tb.Th, Tb.N, Tb.Sp in spines of 3-month-old females (a) *Col1a1-miR-23a* decoy (23D; N=7 for WT, 11 for 23D), **(b)** *Col1a1-miR-27a* decoy (27D; N=8 for WT, 11 for 27D) and **(c)** *Col1a1-miR-24-2* decoy mice (24D; N=10 for WT, 7 for 24D)

Statistical analyses used t-test and results are shown as mean ± S.D.; ****$P<0.001$.

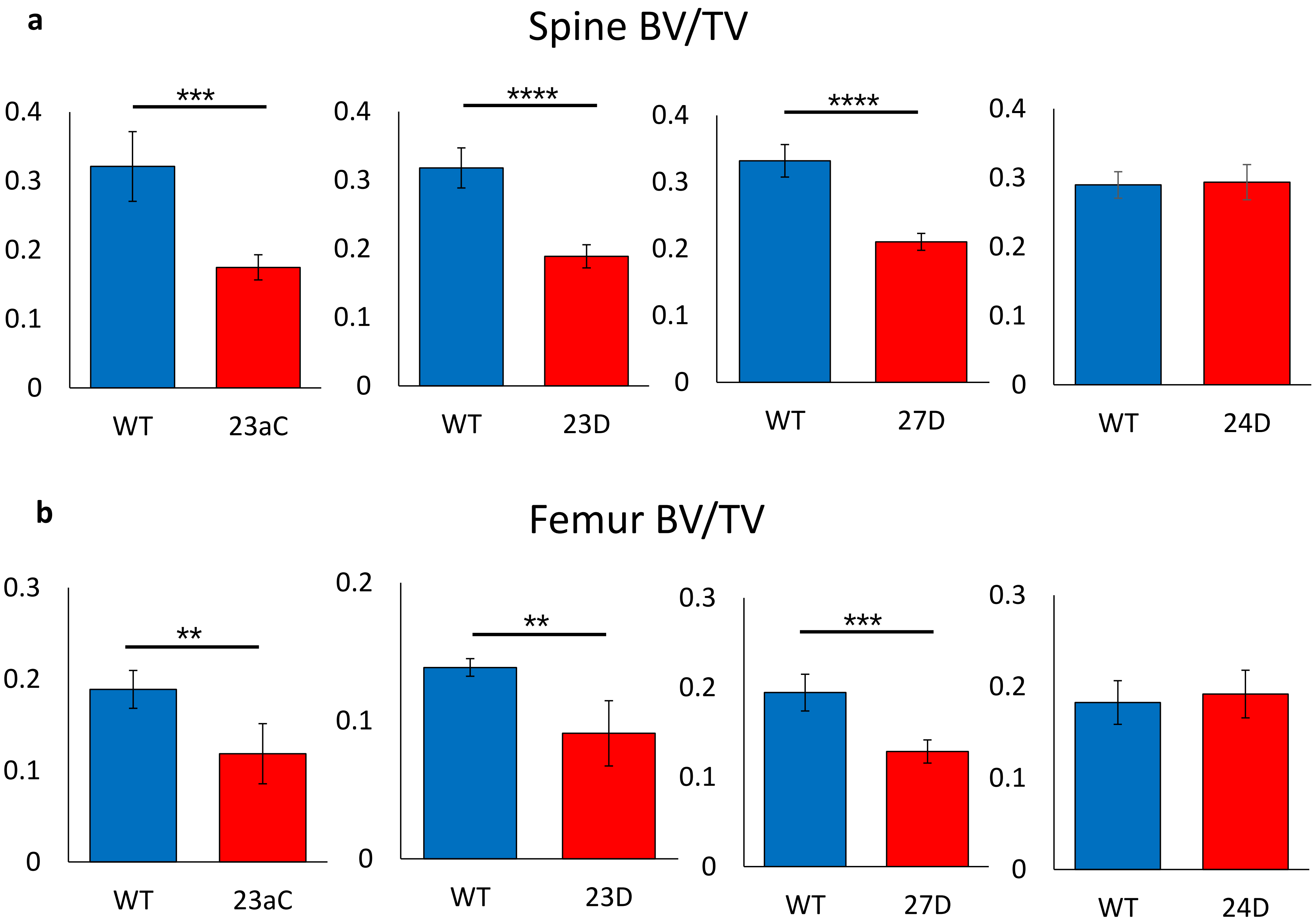
a
Spine BV/TV
***
****
****
0.4
0.3
0.2
0.1
0
WT
23aC
23D
27D
24D
b
Femur BV/TV
**
**
***
0.3
0.2
0.1
0
WT
23aC
23D
27D
24D

Supplementary Figure 6 | μCT analysis of the independent transgenic mouse lines.

μCT analysis of BV/TV in **(a)** spines and **(b)** femurs of 3-month-old female *Col1a1-miR-23aC* (23aC), *Col1a1-miR-23a* decoy (23D), *Col1a1-miR-27a* decoy (27D) and *Col1a1-miR-24-2* decoy (24D) mice (N=4 for each group).

Statistical analyses used t-test and results are shown as mean ± S.D.; **P<0.01, ***P<0.005, ****P<0.001.

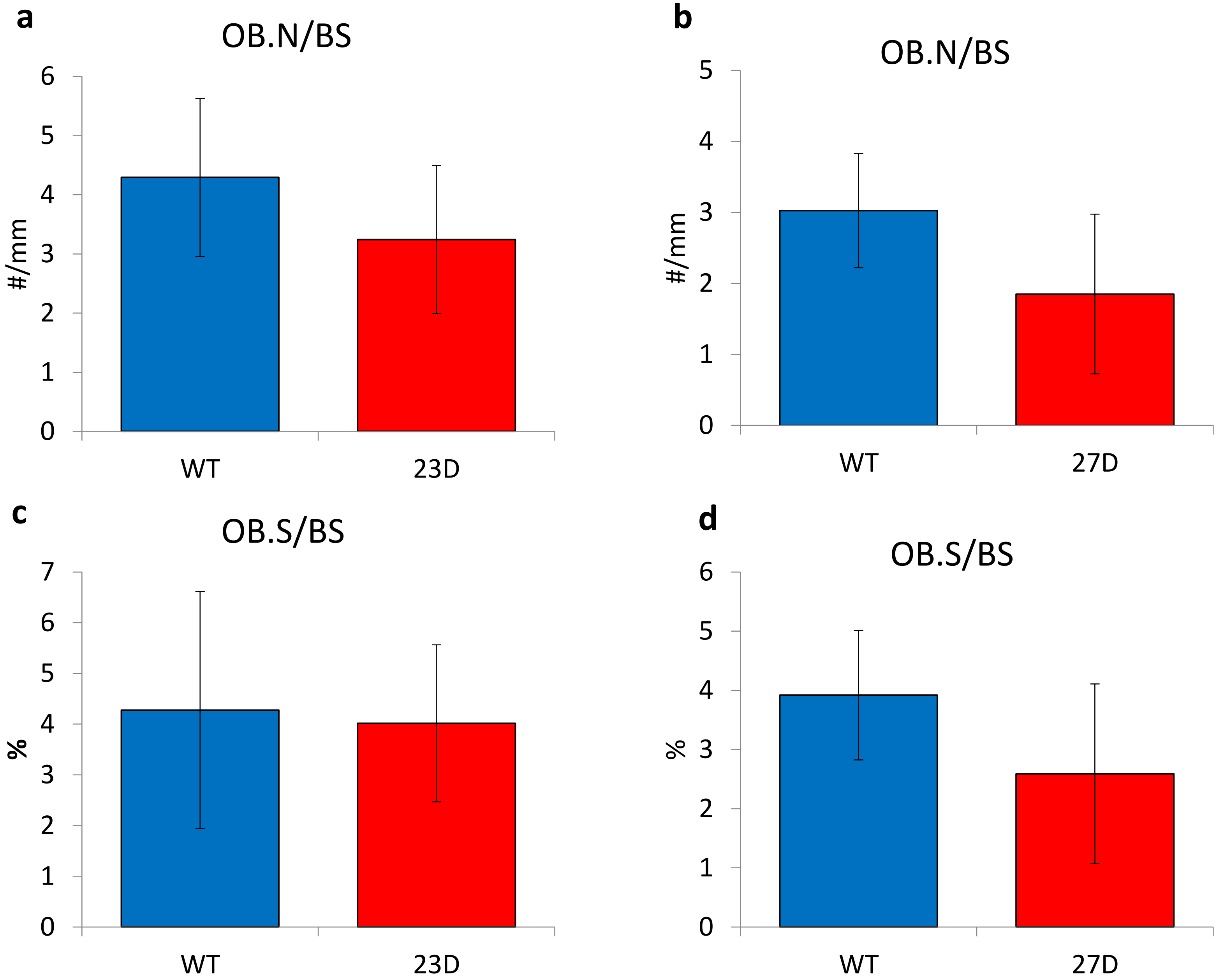
a
OB.N/BS
#/mm
0
1
2
3
4
5
6
WT
23D
b
OB.N/BS
#/mm
0
1
2
3
4
5
WT
27D
c
OB.S/BS
%
0
1
2
3
4
5
6
7
WT
23D
d
OB.S/BS
%
0
1
2
3
4
5
6
WT
27D

Supplementary Figure 7 | Osteoblast parameters in spine trabecular bones were not significantly changed in LOF mice.

Osteoblast number/bone surface (OB.N/BS) was not significantly changed in **(a)** *Col1a1-miR-23a* decoy (23D) and **(b)** *Col1a1-miR-27a* decoy (27D) mice. Osteoblast surface/bone surface (OB.S/BS) was not significantly changed in **(c)** *Col1a1-miR-23a* decoy (23D) and **(d)** *Col1a1-miR-27a* decoy (27D) mice (N=7 for each group).

Statistical analyses used t-test and results are shown as mean ± S.D.; no statistically significant difference was found for any mutant line.

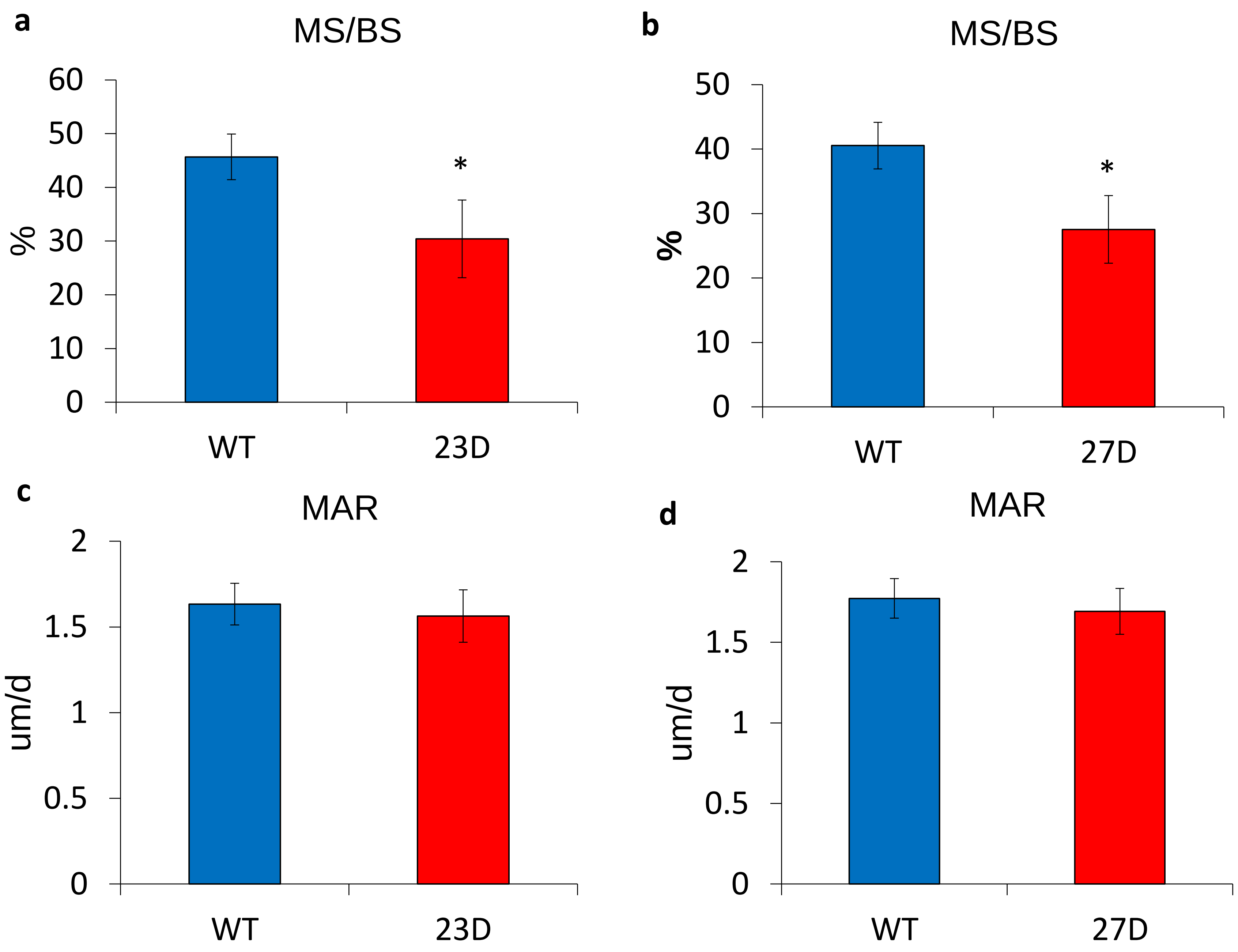
a
MS/BS
%
60
50
40
30
20
10
0
*
WT
23D
b
MS/BS
%
50
40
30
20
10
0
*
WT
27D
c
MAR
um/d
2
1.5
1
0.5
0
WT
23D
d
MAR
um/d
2
1.5
1
0.5
0
WT
27D

Supplementary Figure 8 | Mineral surface/bone surface in spine trabecular bones was significantly decreased in LOF mice.

Mineral surface/bone surface (MS/BS) was significantly decreased in **(a)** *Col1a1-miR-23a* decoy (23D) and **(b)** *Col1a1-miR-27a* decoy (27D) mice. But mineral apposition rate (MAR) was not significantly changed in **(c)** *Col1a1-miR-23a* decoy (23D) and **(d)** *Col1a1-miR-27a* decoy (27D) mice (N=7 for each group).

Statistical analyses used t-test and results are shown as mean ± S.D.; *P<0.05.

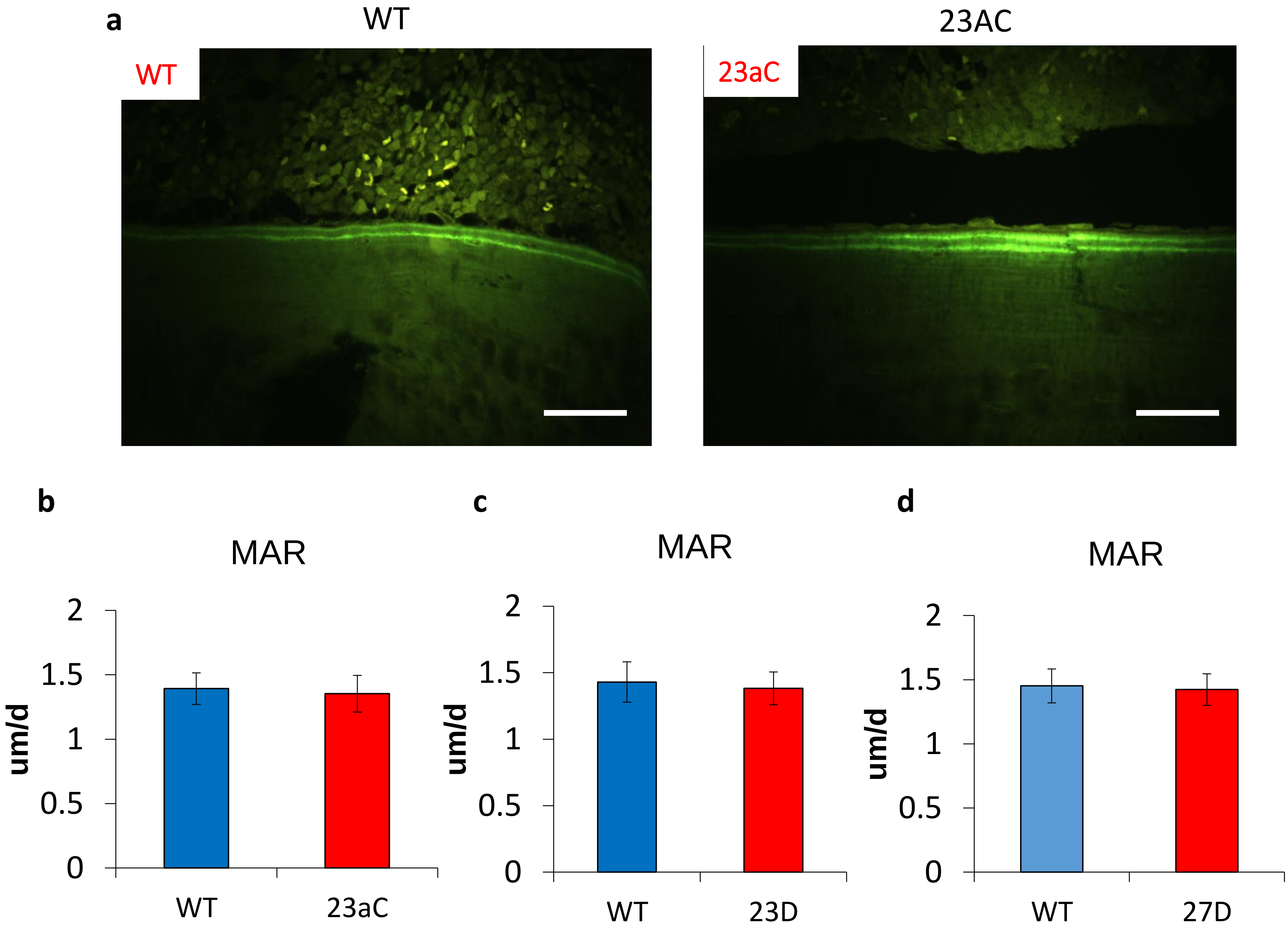
a
WT
23AC
WT
23aC
b
MAR
um/d
2
1.5
1
0.5
0
WT
23aC
c
MAR
um/d
2
1.5
1
0.5
0
WT
23D
d
MAR
um/d
2
1.5
1
0.5
0
WT
27D

Supplementary Figure 9 | Mineral apposition rate in femur cortical bones was not significantly changed in GOF and LOF mice.

a. Representative images of calcein double labeling showing mineralization on the endosteal surface of femur cortical bones of *Col1a1-miR-23aC* (23aC) mice and wild-type littermates (WT). White bars indicate scale of 100 μm.

Quantification of mineral apposition rate (MAR) in **(b)** Col1a1-miR-23aC (23aC), **(c)** Col1a1-miR-23a decoy (23D), **(d)** Col1a1-miR-27a decoy (27D) mice showed no significant difference between groups.

Statistical analyses used t-test and results are shown as mean ± S.D.; no statistically significant difference was found for any mutant line.

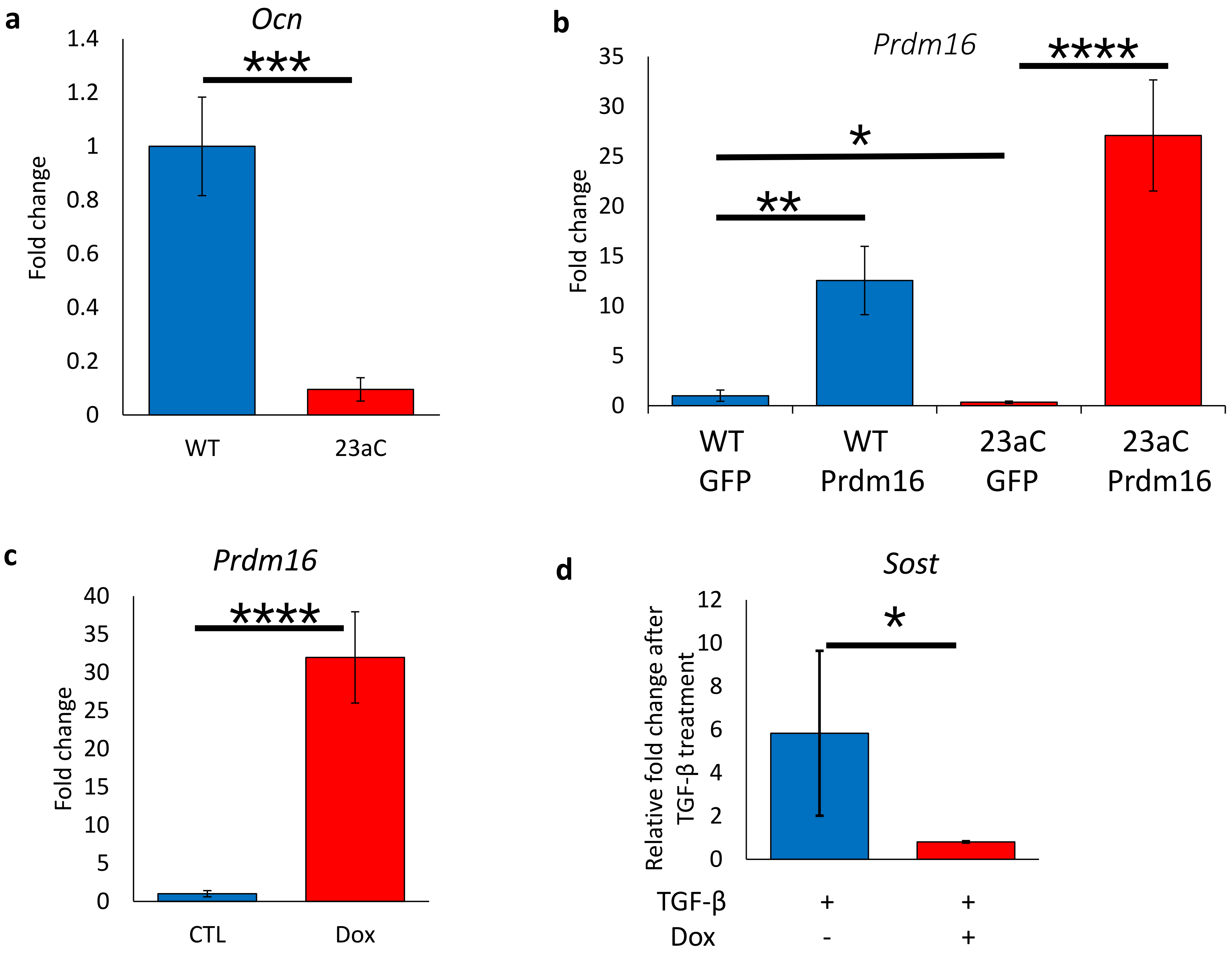
a
Ocn
***
Fold change
1.4
1.2
1
0.8
0.6
0.4
0.2
0
WT
23aC
b
Prdm16
****
*
**
Fold change
35
30
25
20
15
10
5
0
WT GFP
WT Prdm16
23aC GFP
23aC Prdm16
c
Prdm16
****
Fold change
40
35
30
25
20
15
10
5
0
CTL
Dox
d
Sost
*
Relative fold change after TGF-β treatment
12
10
8
6
4
2
0
TGF-β + +
Dox - +

Supplementary Figure 10 | Gene expression profiles in the *in vitro* assays.

a. Expression level of Osteocalcin (*Ocn*) in in differentiation of bone marrow stromal cells (BMSCs) from WT or *Col1a1-miR-23aC* (23aC) mice at day 14 (N=3).

b. Expression levels of *Prdm16* in BMSCs from WT or *Col1a1-miR-23aC* (23aC) mice transduced with GFP- or *Prdm16*-expressing lentivirus at day 28 of differentiation (N=3).

c. Expression levels of *Prdm16 in* an MC3T3-E1 stable cell line transduced with Prdm16-expressing lentivirus. The expression can be induced by doxycycline (1uM) in 48 hours. CTL, control without doxycycline; Dox, doxycycline (N=3).

d. The relative expression levels of *Sost* in this MC3T3-E1 stable cell line were increased after TGF-β treatment (10 ng/mL) for 24 hours. This increase was suppressed with *Prdm16* induction by doxycycline (1uM, 24hours prior to TGF-β treatment). CTL, control without doxycycline; Dox, doxycycline.

Statistical analyses used t-test and results are shown as mean ± S.D.; *P<0.05, **P<0.01, ***P<0.005, ****P<0.001.

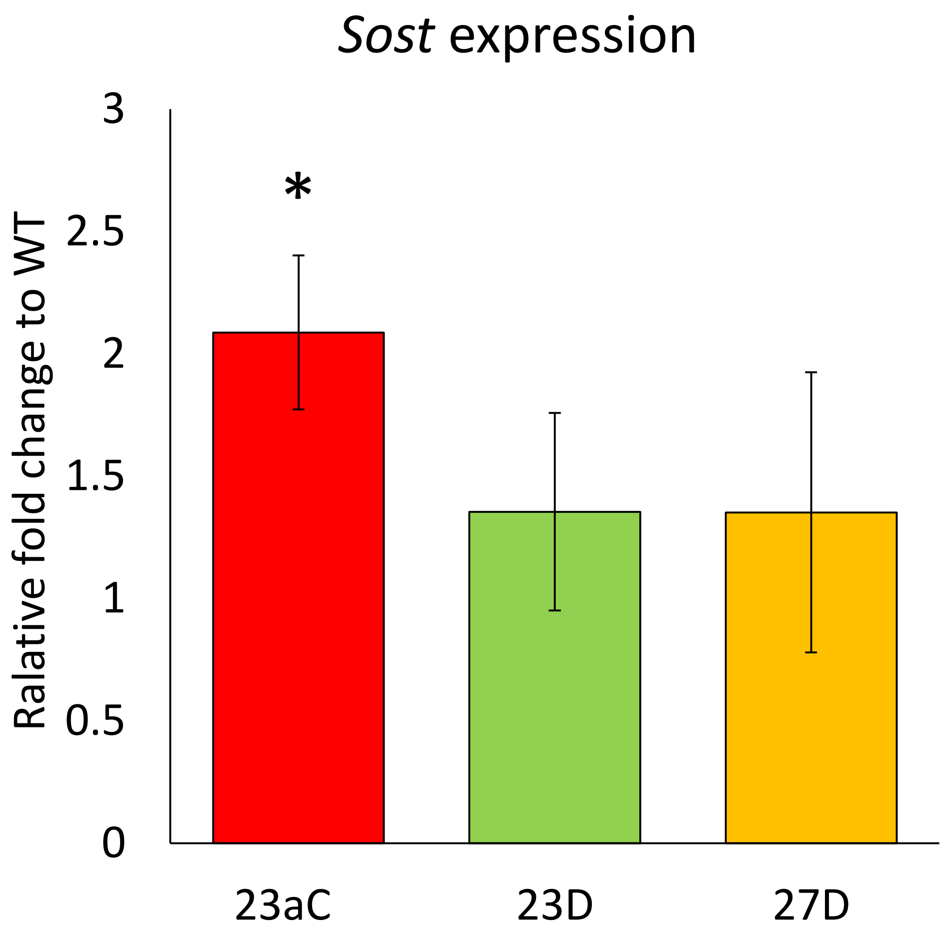
Sost expression
Ralative fold change to WT
3
2.5
2
1.5
1
0.5
0
*
23aC
23D
27D

Supplementary Figure 11 | *Sost* expression in upregulated in bones of GOF mouse model

Expression levels of *Sost* in femurs of 3-month-old mice were measured by real-time PCR. RNA samples from each transgenic mouse line were compared with those from WT littermates (N=4 for each group). Only the expression of *Sost* in GOF mice was significant upregulated.

Statistical analyses used t-test and results are shown as mean ± S.D.; *P<0.05.

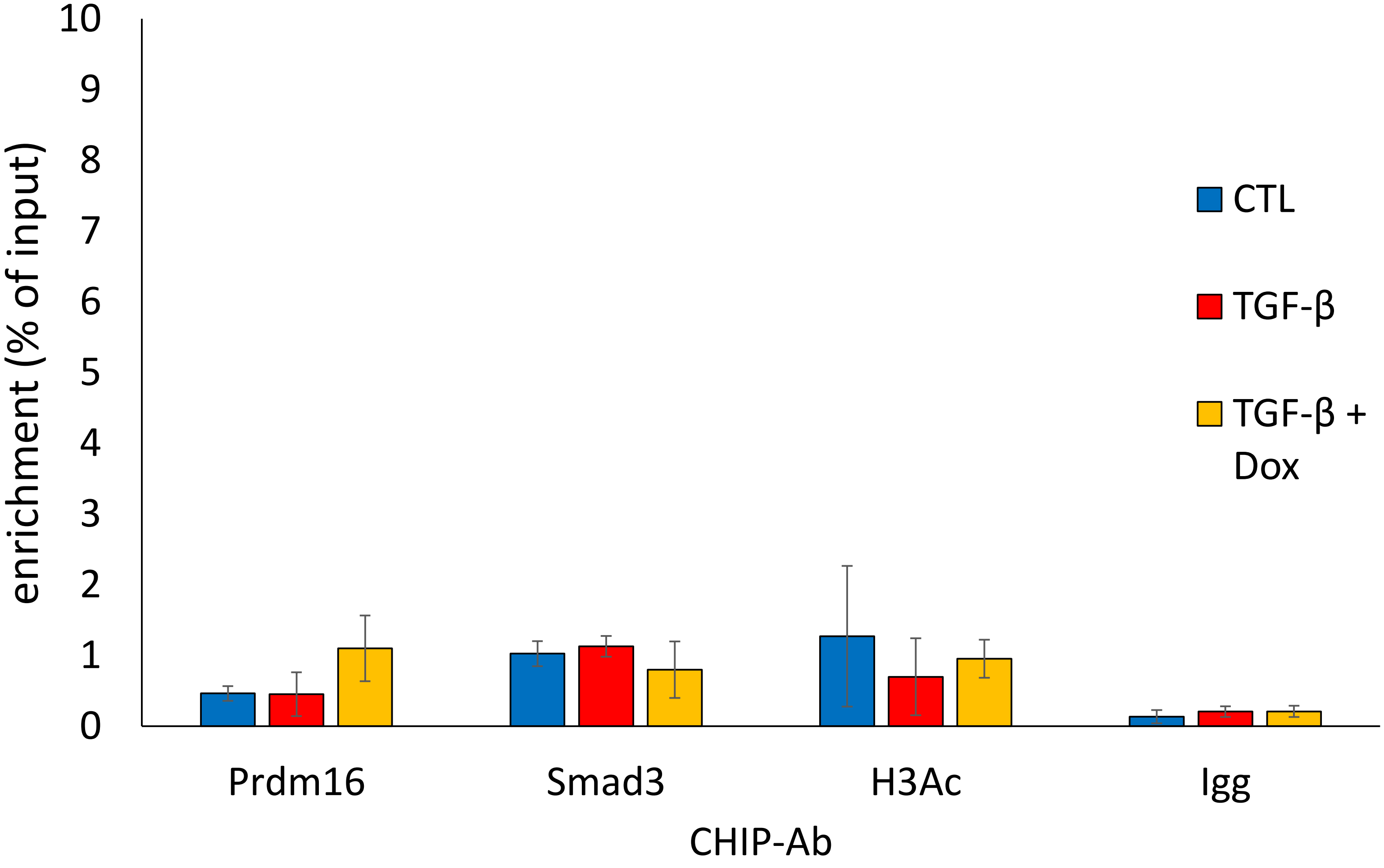

10
9
8
7
6
5
4
3
2
1
0
enrichment (% of input)
CTL
TGF-β
TGF-β + Dox
Prdm16
Smad3
H3Ac
Igg
CHIP-Ab

Supplementary Figure 12 | Negative control region for Chip-PCR assay

The genomic region of Gapdh ‘3UTR is used as a negative control for Chip-PCR assay. Igg is used as a negative control antibody. None of the antibodies showed significant enrichment of the proteins in this study.

Statistical analyses used one-way ANOVA and data are shown as mean ± S.D.; no statistically significant difference was found for any group.

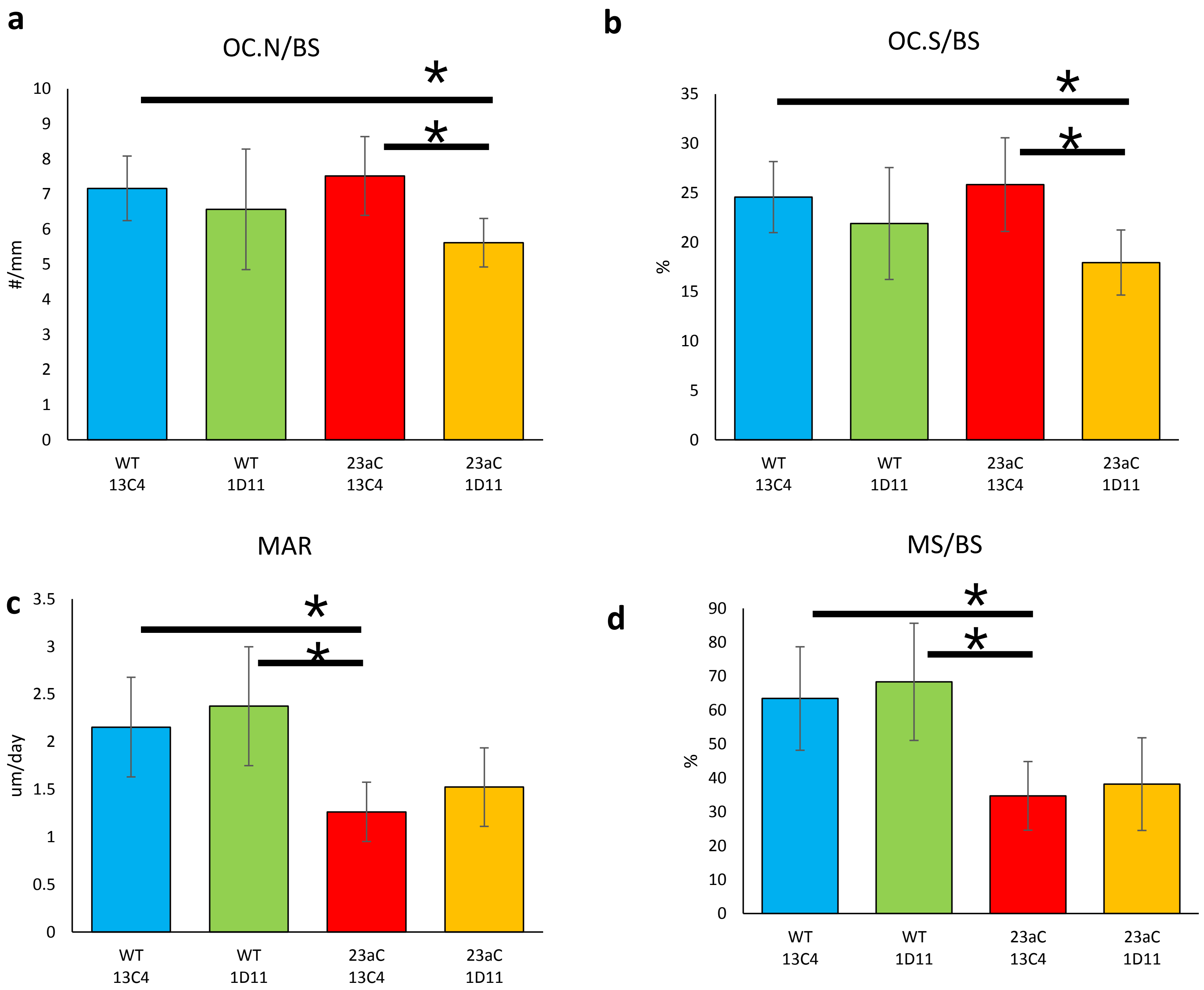

a
OC.N/BS
#/mm
10
9
8
7
6
5
4
3
2
1
0
*
*
WT 13C4
WT 1D11
23aC 13C4
23aC 1D11
b
OC.S/BS
%
35
30
25
20
15
10
5
0
*
*
WT 13C4
WT 1D11
23aC 13C4
23aC 1D11
c
MAR
um/day
3.5
3
2.5
2
1.5
1
0.5
0
*
*
WT 13C4
WT 1D11
23aC 13C4
23aC 1D11
d
MS/BS
%
90
80
70
60
50
40
30
20
10
0
*
*
WT 13C4
WT 1D11
23aC 13C4
23aC 1D11

Supplementary Figure 13 | Osteoclasts were decreased and mineralization was increased in GOF mice by anti-TGF-β antibody.

Bone resorption was assessed by **(a)** osteoclast number (OC.N/BS) and **(b)** osteoclast surface (OC.S/BS) in the spines of *Col1a1-miR-23a* (23aC) mice and wild-type littermate (WT). Bone formation was assessed by **(c)** mineral apposition rate (MAR) and **(d)** mineralization surface/bone surface (MS/BS) in the spines of Col1a1-miR-23a mice and WT littermate (N=4 for each group).

Statistical analyses used one-way ANOVA and data are shown as mean ± S.D.; $^*P<0.05$.

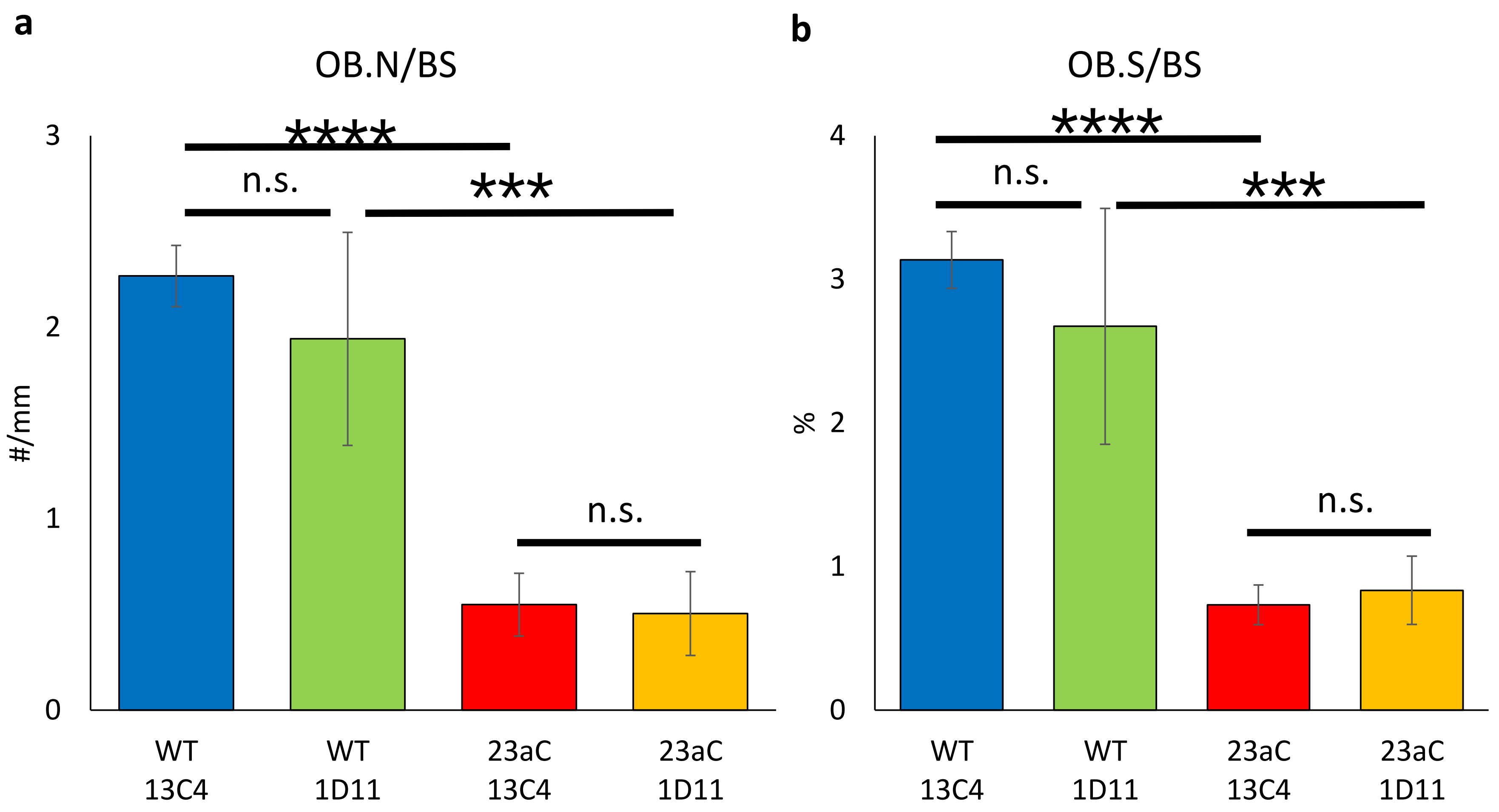
a
OB.N/BS
****
n.s.
***
n.s.
#/mm
3
2
1
0
WT 13C4
WT 1D11
23aC 13C4
23aC 1D11
b
OB.S/BS
****
n.s.
***
n.s.
%
4
3
2
1
0
WT 13C4
WT 1D11
23aC 13C4
23aC 1D11

Supplementary Figure 14 | Osteoblast phenotype was not rescued by anti-TGF-β antibody.

a. Osteoblast number/bone surface (OB.N/BS) and **b**, osteoblast surface/bone surface (OB.S/BS) were significantly decreased in GOF *Col1a1-miR-23a* (23aC) mice groups treated with control antibody (13C4) and anti-TGF-β antibody (1D11) compared to WT groups. There is no significant change between control antibody (13C4) treatment and anti-TGF-β antibody (1D11) treatment (N=4 for each group).

Statistical analyses used one-way ANOVA and data are shown as mean ± S.D.; ***P<0.005, ****P<0.001

Gene ID	FoldChange 23aC/WT	P-val
Col25a1	1.646315465	2.85E-06
Ranbp3l	1.655143673	2.51E-05
Celsr1	1.673339515	2.08E-06
Creb5	1.754236495	1.33E-05
Kcnh2	1.771356161	2.64E-09
Pcdh7	1.78759367	5.53E-16
Fign	1.824322967	4.60E-05
Dmp1	1.831132523	2.10E-18
Frzb	1.842418408	2.36E-10
Hpgd	1.849586962	8.18E-12

Gene ID	FoldChange 23aC/WT	P-val
Col10a1	0.039367187	3.10E-02
Hrc	0.433178558	4.82E-02
Coro6	0.52678846	9.52E-05
Ihh	0.532925485	4.58E-02
Bglap	0.537119965	6.72E-05
Adssl1	0.545801351	3.69E-02
Lipc	0.581623396	2.95E-06
Rbm38	0.594483835	2.65E-02
Scin	0.60395772	1.37E-02
Macrod1	0.611098786	3.45E-02

Supplementary Table 1 | List of the most upregulated and downregulated genes in GOF mice.

This list included 10 most upregulated and 10 most downregulated genes in the differentially expressed genes from GOF RNA-Seq results.

Pathway	Predicted Activation State	Activation z-score	P-value
TGF-β	Activated	2.327	4.75E-06
VEGF	Activated	3.720	3.97E-04
Estrogen Receptor	N/A	-1.807	2.46E-03
P38 MAPK	Activated	2.603	2.75E-03
GM-CSF	N/A	0.640	5.58E-03

Supplementary Table 2 | List of the most affected signaling pathways in GOF mice.

This list includes 5 affected signaling pathways predicted by IPA analysis in GOF RNA-Seq results.
